# Functional Gene Polymorphisms in the Serotonin System and Traumatic Life Events Modulate the Neural Basis of Fear Acquisition and Extinction

**DOI:** 10.1371/journal.pone.0044352

**Published:** 2012-09-05

**Authors:** Andrea Hermann, Yvonne Küpper, Anja Schmitz, Bertram Walter, Dieter Vaitl, Jürgen Hennig, Rudolf Stark, Katharina Tabbert

**Affiliations:** 1 Department of Psychotherapy and Systems Neuroscience, Justus Liebig University Giessen, Giessen, Germany; 2 Bender Institute of Neuroimaging, Justus Liebig University Giessen, Giessen, Germany; 3 Department of Psychology, Section Differential Psychology and Personality Research, Justus Liebig University Giessen, Giessen, Germany; University of Sydney, Australia

## Abstract

Fear acquisition and extinction are crucial mechanisms in the etiology and maintenance of anxiety disorders. Moreover, they might play a pivotal role in conveying the influence of genetic and environmental factors on the development of a (more or less) stronger proneness for, or resilience against psychopathology. There are only few insights in the neurobiology of genetically and environmentally based individual differences in fear learning and extinction. In this functional magnetic resonance imaging study, 74 healthy subjects were investigated. These were invited according to 5-HTTLPR/rs25531 (S+ vs. L_A_L_A_; triallelic classification) and TPH2 (G(-703)T) (T+ vs. T-) genotype. The aim was to investigate the influence of genetic factors and traumatic life events on skin conductance responses (SCRs) and neural responses (amygdala, insula, dorsal anterior cingulate cortex (dACC) and ventromedial prefrontal cortex (vmPFC)) during acquisition and extinction learning in a differential fear conditioning paradigm. Fear acquisition was characterized by stronger late conditioned and unconditioned responses in the right insula in 5-HTTLPR S-allele carriers. During extinction traumatic life events were associated with reduced amygdala activation in S-allele carriers vs. non-carriers. Beyond that, T-allele carriers of the TPH2 (G(−703)T) polymorphism with a higher number of traumatic life events showed enhanced responsiveness in the amygdala during acquisition and in the vmPFC during extinction learning compared with non-carriers. Finally, a combined effect of the two polymorphisms with higher responses in S- and T-allele carriers was found in the dACC during extinction. The results indicate an increased expression of conditioned, but also unconditioned fear responses in the insula in 5-HTTLPR S-allele carriers. A combined effect of the two polymorphisms on dACC activation during extinction might be associated with prolonged fear expression. Gene-by-environment interactions in amygdala and vmPFC activation may reflect a neural endophenotype translating genetic and adverse environmental influences into vulnerability for or resilience against developing affective psychopathology.

## Introduction

More than 25% of the western population develops an anxiety or mood disorder at least once in a lifetime [1]. Contemporary learning theories highlight the role of stressful or traumatic life events in the etiology of these disorders [2], [3], with classical fear conditioning being a central mechanism for the acquisition of fear in response to innocuous stimuli [4], [5]. The amygdala, insula, and dorsal anterior cingulate cortex (dACC) have been identified as important brain structures underlying the acquisition and expression of conditioned fear [5], [6]. Beyond that, difficulties in the ability to diminish or extinguish conditioned responses are probably highly relevant in the development and maintenance of anxiety disorders [7]. In particular, the ventromedial prefrontal cortex (vmPFC) has been shown to be involved in extinction learning and recall [8], [9].

Yet, not every individual encountering a highly stressful situation develops an anxiety or mood disorder [2], [4]. These individual differences may in part be genetically driven and/or based on prior learning experiences including traumatic life events [10]. Genetic association studies are one possible strategy to study genetically based individual differences. Genetic polymorphisms within the serotonergic (5-HT) system are especially promising candidates for these studies, because of 5-HT’s crucial involvement in the development of mental disorders [11]. Several genetic polymorphisms known to account for variability in presynaptic serotonergic neurotransmission have been identified. One of the most well-known is the serotonin transporter gene linked polymorphic region (5-HTTLPR), with a short variant (S-allele) comprising 14 copies of a 20–23 base pair repeat and a long variant (L-allele) comprising 16 copies. The S-allele has been related to reduced presynaptic 5-HT reuptake, increased trait negative affect [12], and stronger neural responses (e.g., in the amygdala) towards emotional stimuli [13]–[15]. There are only few studies examining the influence of the 5-HTTLPR on psychophysiological correlates of fear conditioning [16]–[19] (for an overview see [20]), which taken together indicate stronger fear conditioning in S-allele carriers compared with non-carriers. In a first study, Garpenstrand and colleagues [16] found an over-representation of the 5-HTTLPR S-allele in 20 individuals that showed good acquisition compared with 20 individuals that showed bad acquisition of conditioned SCRs (preselected from a group of 346 fear conditioned individuals). Furthermore, these S-allele carriers were characterized by stronger fear conditioned SCRs compared with non-carriers. Enhanced startle potentiation but not skin conductance responding to the conditioned stimulus (CS+) has been observed in S-allele carriers compared with non-carriers during fear conditioning [17]. There is one investigation demonstrating stronger differential fear conditioned neural responses in the right amygdala, bilateral insula, left thalamus and bilateral occipital cortex in SS-homozygotes compared with heterozygotes/non-carriers [18]. In addition, observational fear learning has been shown to be associated with enhanced conditioned skin conductance responses in S-allele-carriers [19]. There is also one study that utilized an instructed fear paradigm showed that S-allele carriers compared with LL-homozygotes exhibit stronger fear potentiated startle responses [21].

In addition to these studies on fear conditioning and negative affect, a recent review article indicates that carriers of the S-allele are probably characterized by an overall increased responsiveness to external cues regardless of their valence [11]. In accordance with this hypothesis, one study reports stronger acquisition of appetitive conditioned responses (using erotic pictures as unconditioned stimuli) in the amygdala, insula, thalamus and orbitofrontal cortex in S-allele carriers compared with non-carriers, and in the ventral striatum in SS-homozygotes compared with heterozygotes/non-carriers [22].

Previous research also suggests that S-allele carriers, who were exposed to a high number of traumatic life events, show an increased risk for depression [3], [23], enhanced cortisol responses to stress [24], an altered resting activation and functional connectivity of amygdala and hippocampus [13], and stronger conditioned responses in the insula and occipital cortex [18]. However, recent meta-analyses have severely challenged the validity of the reported gene-environment interactions concerning an increased risk for depression [25], [26]. Some of these reported inconsistencies regarding the 5-HTTLPR may be due to the effects of a single nucleotide polymorphism (rs25531, A/G SNP) within the 5-HTTLPR sequence, which has been shown to influence m-RNA expression of the *5-HT transporter gene* [27]. Only the L_A_-allele is associated with high 5-HTT mRNA levels, while the L_G_-allele seems to be functionally equivalent to the low-expressing S-allele.

Another widely studied serotonergic polymorphism is the single nucleotide polymorphism (SNP) rs4570625 (G(−703)T) in the promoter region of the *tryptophan hydroxylase-2 (TPH2) gene* influencing the rate of 5-HT synthesis in the presynapse [28]. Even though no direct effect on TPH2 expression rates has been shown for the rs4570625 polymorphism, the SNP is part of the same haplotype block as a SNP (rs11178997) which does affect TPH2 expression rates [29]. Taken together these data tentatively suggest that the T-allele of the rs4570625 might be indirectly linked to a reduced promoter activity. The T-allele has also been associated with enhanced harm avoidance scores in healthy individuals [30], cluster B and C personality disorders in patients [28], and enhanced neural (e.g., amygdala) responses to emotional stimuli [31], [32]. Furthermore, some studies highlight the importance of gene-environment interactions [33]–[35].

Recent studies report combined effects of the 5-HTTLPR and the TPH2 (G(−703)T) polymorphisms on event-related potentials [36] and neural (e.g., amygdala) activation during emotional stimulation [37]. In these studies, strongest responses were observed in carriers of both the S- and T-allele and least responses in LL- and GG-homozygotes, probably demonstrating the interplay of different genetic factors.

Despite the importance of both, emotional learning processes, as well as serotonergic polymorphisms and their interaction with environmental factors in the development of psychiatric disorders, there is only one study so far that investigates the influence of the 5-HTTLPR polymorphism and its interaction with traumatic life events on neural correlates of fear acquisition [18]. However, no study to date examined the influence of the 5-HTTLPR and its interaction with traumatic life events on neural correlates of fear extinction, or the influence of the TPH2 (G(−703)T) polymorphism on the neural basis of fear conditioning. In this functional magnetic resonance imaging (fMRI) study we therefore assessed genetic (5-HTTLPR and TPH2 (G(−703)T)) and environmental (traumatic life events) effects on amygdala, insula, dACC and vmPFC activation during the acquisition and extinction of conditioned fear in a well-established differential fear conditioning paradigm. It was hypothesized, that T- and/or S-allele carriers and especially those who experienced a higher number of traumatic life events, show stronger acquisition of electrodermal and neural (e.g., in the amygdala, insula and dACC) conditioned fear responses. These responses might manifest as stronger early acquisition, stronger fear expression after initial learning (late acquisition phase), or prolonged fear expression during fear extinction. Moreover, these individuals are assumed to show altered fear extinction mechanisms reflected in altered differential amygdala and vmPFC activation. However, the direction of these altered responses remains speculative.

## Materials and Methods

### Ethics Statement

All subjects gave written informed consent. Both aspects of the study (molecular genetics and fMRI) were approved by the ethics committee of the German Psychological Society (DGPs).

### Subjects and Questionnaires

Seventy-eight Caucasian subjects were invited according to TPH2 (G(−703)T) (T+ vs. T−) and 5-HTTLPR/rs25531 (referred to as 5-HTTLPR polymorphism; S+ vs. L_A_L_A_; triallelic classification) genotype from an existing data base. This results in 4 genotype groups (T+S+ (n = 20), T+S− (n = 18), T−S+ (n = 20), T−S− (n = 20)). The original pool of subjects consisted of 742 persons (397 women and 345 men). Genotype distribution did not deviate significantly from Hardy–Weinberg equilibrium for either polymorphism (TPH2 (G(−703)T): *χ*2 = 1.021, *p>.*05; 5-HTTLPR/rs25531: *χ*2 = 1.82, *p>.*05).

Depressive symptoms were assessed with the Beck Depression Inventory II (BDI II; [38]), trait anxiety with the State-Trait-Anxiety-Inventory (STAI; [39]), and present and past psychopathology with a short clinical interview. Due to clinically relevant depression scores (BDI II score >18), three subjects from the T−S+ group were excluded. One further subject from the T+S− group was excluded due to excessive head movements during scanning. Thus, the final sample consisted of 74 subjects (37 males/37 females; age: 19–41 years, *M* = 24.38, *SD* = 4.14; see Table 1 for further characteristics of the final sample). None of the subjects had a history of any psychiatric or neurological treatment (including psychopharmacological medication), and all of the participants were right-handed.

**Table 1:** Sample description.

	T+S+	T+S-	T-S+	T-S-	Test forgroup differences
**TPH2 (G(**−**703)T)/rs4570625**	TT, GT	TT, GT	GG	GG	–
**5-HTTLPR/rs25531**	SS, SL_G_, L_G_L_G_, SL_A_, L_G_L_A_	L_A_L_A_	SS, SL_G_, L_G_L_G_, SL_A_, L_G_L_A_	L_A_L_A_	–
**n**	20	17	17	20	–
**Sex (F/M)**	10/0	9/8	8/9	10/10	*χ^2^* = .12, *p* = .99
**Age** (mean [SD])	24.80 (4.25)	23.00 (2.81)	25.47 (5.16)	24.20 (3.86)	*F* = 1.13, *p* = .35
**BDI-II** (mean [SD])	6.00 (4.38)	5.35 (3.48)	5.47 (5.20)	4.70 (3.23)	*F* = 0.34, *p* = .80
**STAI-T** (mean [SD])	34.65 (5.75)	34.24 (5.37)	33.60 (6.71)	34.80 (6.28)	*F* = 0.13, *p* = .94
**Traumatic life events**	1.10 (1.02)	1.47 (1.84)	1.12 (0.99)	1.35 (1.98)	*F* = 0.25, *p* = .86
(LEC; mean [SD]; [range])	[0–3]	[0–5]	[0–3]	[0–8]	
**Traumatic life events** (LEC; numberof participants with no/one/morethan one traumatic life event)	7/6/7	8/3/6	6/4/7	8/7/5	^1^ *p* = .90

BDI-II: Beck Depression Inventory II [38]. STAI-T: trait scale of the State-Trait-Anxiety-Inventory [39]. LEC: Life Events Checklist [40]. Test for differences between groups: Chi-squared test, one way analysis of variance and Fisher’s exact test^1^.

In order to asses the number of previous traumatic life events (TLE), all subjects answered the Life Events Checklist (LEC, [40]), a 17-item self-report measure capturing multiple types of previous (lifetime) exposure to a wide variety of potentially traumatic experiences (e.g., motor vehicle accident, physical and sexual assault, combat, sudden unexpected death of a loved one). Each item had to be answered by means of a multiple-choice 5-point nominal scale (1 =  happened to me, 2 =  witnessed it, 3 =  learned about it, 4 =  not sure; 5 =  does not apply). In accordance with the initial publication properties [40], total scores for the amount of TLEs in the present study included only TLEs that happened to the participants. The whole group showed a median of 1 (range 0–8). To account for outliers in the number of TLEs the final TLE score for analyses in this study consisted of three levels (instead of the absolute number of TLEs): 0 =  ‘no TLE experienced’, 1 =  ‘one TLE experienced’, 2 =  ‘more than one TLE experienced’ (see Table 1 for distribution by genotype group).

### Genotyping

DNA was extracted from buccal cells and purification of genomic DNA was performed using a MagnaPure system (Roche, Germany) with a standard commercial extraction kit (MagNA Pure LC DNA Isolation Kit I; Roche Diagnostics, Mannheim, Germany).

Genotyping of 5-HTTLPR/rs25531 was carried out as described by Alexander and colleagues [24]. TPH2 (G(−703)T) genotyping was performed by means of real-time PCR (Light Cycler System, Roche, Germany) using melting-curve detection analysis as described previously [30].

### Conditioned Visual Stimuli

Two simple geometric figures, a rhombus and a square, served as CS+ and CS− in a differential conditioning paradigm. The stimuli were grey in color and had identical luminance. Figures were presented for 8 s. For visual stimulation inside the scanner, an LCD-projector was used, which projected pictures onto a screen at the end of the scanner (visual field  = 18°). Pictures were viewed by means of a mirror mounted to the head coil.

### Unconditioned Stimulus

A custom-made impulse-generator (833 Hz) provided transcutaneous electrical stimulation to the left shin through two Ag/AgCl electrodes (0.1 cm^2^ surface each). The electrical stimulation served as the UCS. Stimulus intensity was set to an “unpleasant but not painful” level for each subject individually using a gradually increasing rating procedure. The genotype groups did not differ in UCS intensity after calibration (all p>.2). During the conditioning procedure, each electrical stimulus was applied for 100 ms. Onset and duration of the electrical stimulation were set by a computer program and the impulse-generator inside the scanning chamber was triggered via an optic fiber cable.

### Conditioning Procedure

The conditioning experiment consisted of one session including acquisition and extinction trials. For each participant, there was one acquisition phase with 20 trials of CS+ and CS- respectively. The onset of the UCS-presentation was delayed 7.9 s after each CS+ onset and co-terminated with CS+ presentation (delay-conditioning). The reinforcement rate was 100%. Directly following these acquisition trials, 15 presentations of unpaired CS+ and 15 unpaired CS− presentations were shown (extinction phase). The inter-trial intervals ranged from 9.75 to 14.25 s. One of two pseudo randomized stimulus orders was used comprising the following restrictions: no more than two consecutive presentations of the CS and an equal number of CS+ and CS− trials within 10 trials (five each). The acquisition procedure started with a CS+ for half of the subjects, with a CS− for the other half, and either the rhombus or the square served as CS+.

Directly after the experiment (after the extinction phase), subjects indicated the CS-UCS-contingencies for the CS in three steps. The first step consisted of a free verbal report on the estimated aim of the study. Secondly, participants had to indicate the contingencies in a short recognition questionnaire (“the electrical stimulation followed this stimulus:” “always”, “sometimes”, “never”, “don’t know”). Finally, in a forced choice questionnaire one of the two CS had to be chosen as the stimulus preceding electrical stimulation. All 74 subjects were contingency aware in at least one of the three applied measures.

### Magnetic Resonance Imaging

Brain images were acquired using a 1.5 Tesla whole-body tomograph (Siemens Symphony with a quantum gradient system) with a standard head coil. For functional imaging, a total of 573 volumes were registered using a T2*-weighted gradient echo-planar imaging sequence (EPI) with 25 slices covering the whole brain (slice thickness  = 5 mm; 1 mm gap; descending acquisition order; TA = 100 ms; TE = 55 ms; TR = 2.5 s; flip angle  = 90°; field of view  = 192 mm×192 mm; matrix size  = 64×64). The first three volumes were discarded due to an incomplete steady state of magnetization. In order to keep susceptibility artifacts in the OFC and the amygdala to a minimum, the orientation of the axial slices was parallel to the OFC tissue – bone transition. A gradient echo field map sequence was measured before the functional run to get information for unwarping B_0_ distortions. Furthermore, an anatomical scan (MPRAGE) was conducted to get highly resolved structural information for the normalization procedure. Data were analyzed using Statistical Parametric Mapping (SPM8, Wellcome Department of Cognitive Neurology, London, UK; 2009) implemented in MatLab R2007b (Mathworks Inc., Sherborn, MA). Unwarping and realignment (b-spline interpolation), slice time correction and normalization to the standard space of the Montreal Neurological Institute brain (MNI-brain) were performed. Smoothing was executed with an isotropic three dimensional Gaussian filter with a full width at half maximum (FWHM) of 9 mm.

For each subject acquisition (first and second half) and extinction were integrated in one first level general linear model, resulting in the following regressors: CS+_early acquisition_ (trials 1–10), CS−_early acquisition_ (trials 1–10), CS+_late acquisition_ (trials 11–20), CS−_late acquisition_ (trials 11–20), CS+_extinction_, CS−_extinction_, UCS, non-UCS. Non-UCS was defined as the time window during CS− presentation corresponding to the time window of UCS presentation during the CS+ in the acquisition phase. A further regressor contained the presentation of the first two geometrical figures (CS+ and CS−) of the extinction phase. These 9 regressors were modelled by a stick function convolved with the canonical hemodynamic response function (HRF) in the general linear model, without specifically modelling the durations of the events. The six movement parameters of the rigid body transformation applied by the realignment procedure were introduced as covariates in the model. The time series were filtered with a high pass filter (time constant  = 128 s). Contrasts for conditioned responses (CS+ minus CS−) for the three different phases and for unconditioned responses (UCS minus Non-UCS) were calculated.

For group analysis multiple regression models were estimated (separately for the first and second half of acquisition and the extinction phase) with following regressors: one regressor for each genotype group ((T+S+), (T+S−), (T−S+) and (T−S−)), and one (groupwise mean-centered) regressor per genotype group with the TLE scores, resulting in 8 regressors.

At first, analyses of conditioned (CS+ minus CS−) and unconditioned responses (UCS minus Non-UCS) for the whole group (T-contrasts) were done, before testing for the hypothesized comparisons regarding conditioned (CS+ minus CS−) responses (T-contrasts: TLE_(T+)_ minus TLE_(T−)_; TLE_(S+)_ minus TLE_(S−)_; T+ minus T−, S+ minus S−). For extinction learning the corresponding comparisons were tested via F-tests, as the direction was difficult to hypothesize due to a lack of previous results. Furthermore, exploratory analyses of the 5-HTTLPR x TPH2 (G(−703)T) interaction for conditioned responses (CS+ minus CS−) for all three conditioning phases was done (F-test). In order to investigate combined effects of the two polymorphisms, a further regression analysis with one regressor containing the number of T+ and S+ genotypes (0 = (T−S−), 1 = (T+S−) or (T−S+), 2 = (T+S+)) was carried out for each phase (CS+ minus CS−; F-tests). Unconditioned responses (UCS minus Non-UCS) were tested in parallel to the conditioned responses (interaction and main effects, F-tests).

Region of interest (ROI) analyses (amygdala, insula, dACC and vmPFC) were performed using the small volume correction option of SPM8. The amygdala and insula masks for the ROI analyses were maximum probability masks taken from the “Harvard-Oxford cortical and subcortical structural atlases” provided by the Harvard Center for Morphometric Analysis (http://www.cma.mgh.harvard.edu) with a probability threshold at 0.50. The dACC mask consisted of a 10 mm sphere surrounding a peak voxel for fear conditioning-related neural responses in the anterior cingulate/mid-cingulate gyrus (MNI: x = 0, y = 12, z = 36) as indicated in a meta-analysis of fear conditioning studies [6]. The vmPFC mask was created in MARINA [41], consisting of the bilateral medial orbital area of the frontal cortex and the gyrus rectus according to the parcellation of Tzourio-Mazoyer [42]. The significance threshold was set to α = 0.05 on voxel-level, corrected for multiple testing within a ROI (FWE correction using random field theory; [43]).

### Skin Conductance Responses (SCRs)

SCRs were sampled simultaneously with fMRI scans using Ag/AgCl electrodes filled with isotonic (0.05 M NaCl) electrolyte medium and placed hypothenar at the nondominant hand. SCRs were defined in three analysis windows [44]: The maximum response within a time window of 1 to 5 s after the CS onset was counted as the first interval response (FIR), within a time window of 5 to 8.5 s as the second interval response (SIR), and within a time window of 8.5 to 13 s as the unconditioned response (UCR). Conditioned responses were defined as larger response magnitudes in reaction to the CS+ than to the CS- in the FIR and SIR. Data were logarithmically transformed (natural logarithm) in order to attain statistical normality. Statistical comparisons were performed in analogy to the analyses of the fMRI data. Data of 4 subjects were lost due to technical problems leaving 70 subjects in the final SCR analysis.

## Results

### General Unconditioned and Conditioned Responses

General unconditioned and conditioned neural responses during the first and second half of acquisition have been found in all ROIs in the whole group. During extinction learning significant responses emerged in the bilateral insula and the dACC (see Figure S1). Significant conditioned SCRs during early (FIR: *F*(1,62) = 19.72, *p*<.001; SIR: *F*(1,62) = 15.25, *p*<.001) and late acquisition (FIR: *F*(1,62) = 8.54, *p* = .005) and unconditioned SCRs (TIR; first half: *F*(1,62) = 105.12, *p*<.001; second half: *F*(1,62) = 53.36, *p*<.001) were found in the whole group (except SIR during second half of acquisition; F(1,62) = 3.73, *p* = .058), whereas during extinction no significant responses could be observed (all *p*>.3).

### Interaction Effects of the 5-HTTLPR and TPH2 (G(−703)T) Polymorphisms

As assessed in multiple regression analyses no significant 5-HTTLPR x TPH2 (G(−703)T) interaction emerged for conditioned skin conductance and neural responses for each of the three conditioning phases or during processing of the UCS.

### Combined Effects of the 5-HTTLPR and TPH2 (G(−703)T) Polymorphisms

**Figure 1:**
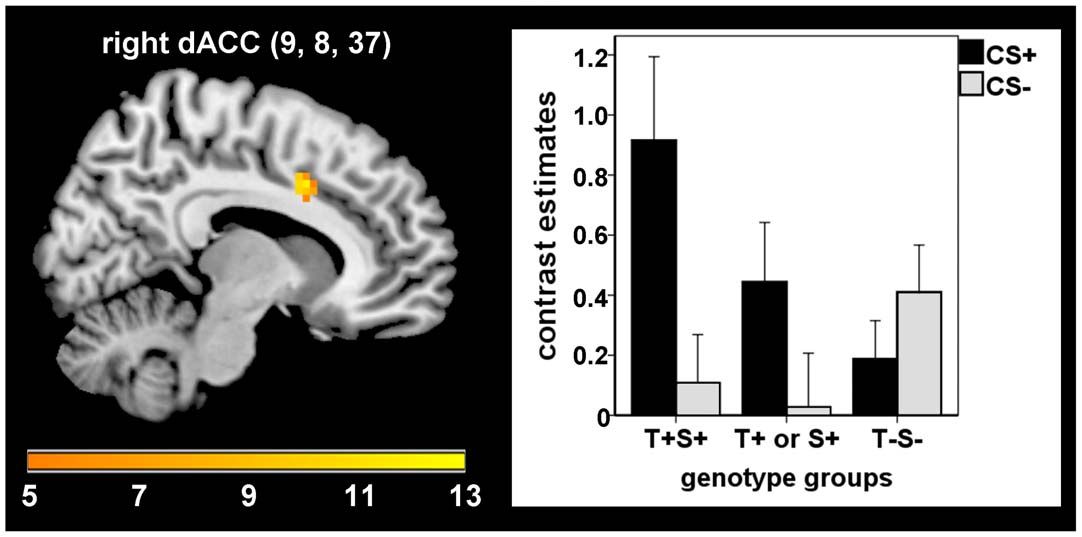
Combined effects of 5-HTTLPR and TPH2 (G(−703)T) genotypes on dACC activation during extinction. Positive association of the number of T+ and/or S+ genotypes (T+S+ gt T+ or S+ gt T−S−) with dorsal anterior cingulate cortex activation during extinction (*F* = 11.11, *p_fwe_* = .047).

During extinction learning combined effects of the two polymorphisms were found in the dACC (MNI: 9, 8, 37; *F*(1,72) = 11.11; *p_fwe_* = .047; Cohen’s *d* = .785), indicating stronger responses in individuals with a higher number of T+ and/or S+ genotypes (see Figure 1). No further associations of (un)conditioned (electrodermal or neural) responses with combined effects of the two studied polymorphisms were found during all learning phases.

### 5-HTTLPR x TLE Interaction

**Figure 2:**
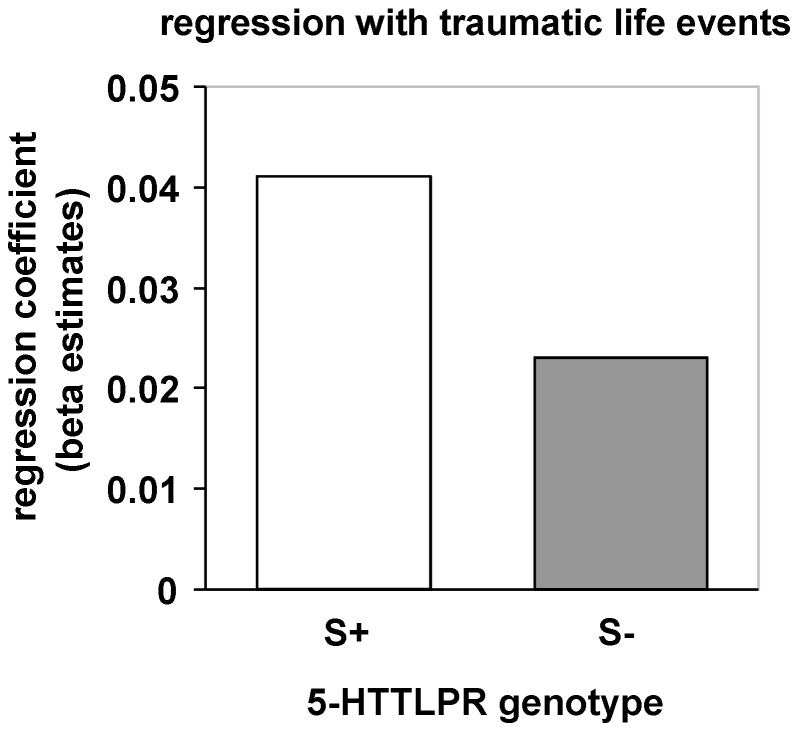
Modulation of conditioned skin conductance responses (SCRs) during late acquisition by 5-HTTLPR genotype and traumatic life events. Stronger positive association of conditioned SCRs during late acquisition in S-allele carriers compared with non-carriers.

Considering conditioned neural responses, no interaction of 5-HTTLPR genotype and the number of previously encountered traumatic life events emerged during early and late acquisition. However, S-allele carriers with a higher number of traumatic life events compared with non-carriers showed stronger electrodermal conditioned responses (FIR) during the second half of acquisition (*F*(1,62) = 4.3, *p* = .042; see Figure 2).

**Figure 3:**
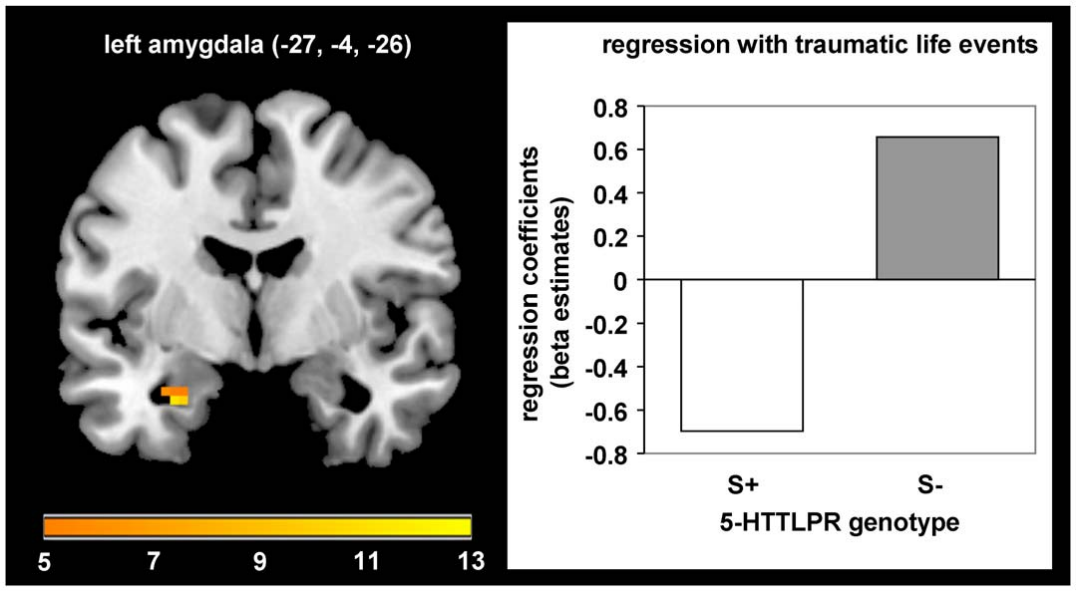
Modulation of left amygdala activation during extinction by *5-HTTLPR* genotype and traumatic life events. Stronger negative association of traumatic life events with left amygdala activation in S-allele carriers compared with non-carriers during extinction (*F* = 12.06, *p_fwe_* = .025).

During extinction learning, an interaction between 5-HTTLPR genotype and traumatic events emerged in the left amygdala (MNI: −27, −4, −26; *F*(1,66) = 12.06; *p_fwe_* = .025, Cohen’s *d* = .854). S-allele-carriers with a higher number of traumatic life events showed reduced activation in the left amygdala compared with non-carriers (see Figure 3).

There were no further significant 5-HTTLPR x TLE interactions in neural and electrodermal (un)conditioned responses.

### Main Effect 5-HTTLPR

**Figure 4:**
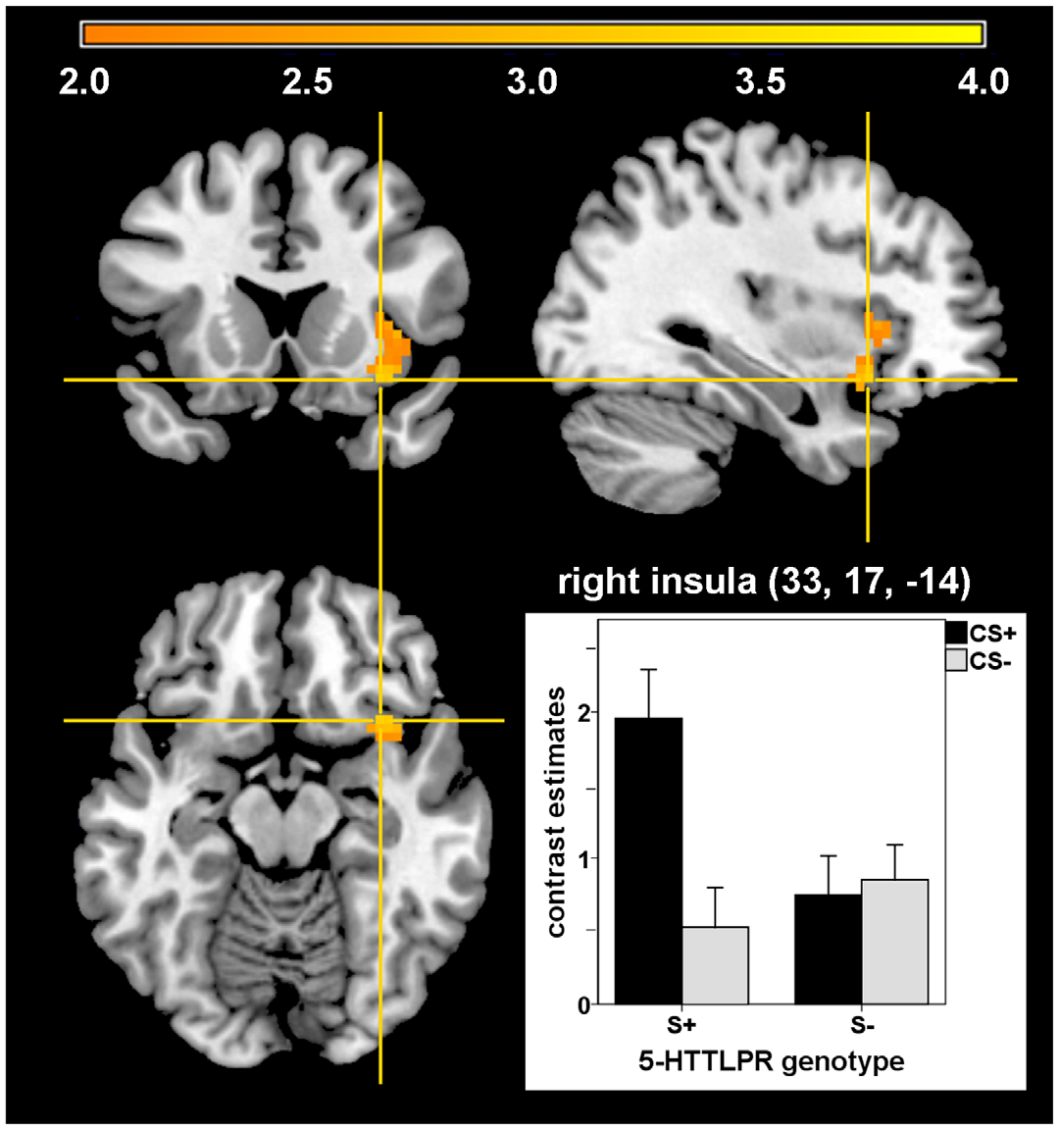
5-HTTLPR polymorphism and right insula activation during late acquisition. Enhanced right insula activation in S-allele-carriers compared with non-carriers in response to the CS+ vs. CS− during late acquisition (*T* = 3.38, *p_fwe_* = .044).

Although we did not find any differences during the first half of acquisition, S-allele carriers revealed stronger activation in the right insula (MNI: 33, 17, −14; *T*(66) = 3.38; *p_fwe_* = .044; Cohen’s *d* = .832) compared with non-carriers during late acquisition in response to the CS+ vs. CS- (see Figure 4). Moreover, S-allele carriers vs. non-carriers showed stronger unconditioned responses in the right insula (MNI: 39, 11, −14; *F*(66) = 12.96; *p_fwe_* = .047, Cohen’s *d* = .886). There was no further main effect of 5-HTTLPR genotype on (un)conditioned neural or skin conductance responses during all three conditioning phases.

### TPH2 (G(−703)T) x TLE Interaction

**Figure 5:**
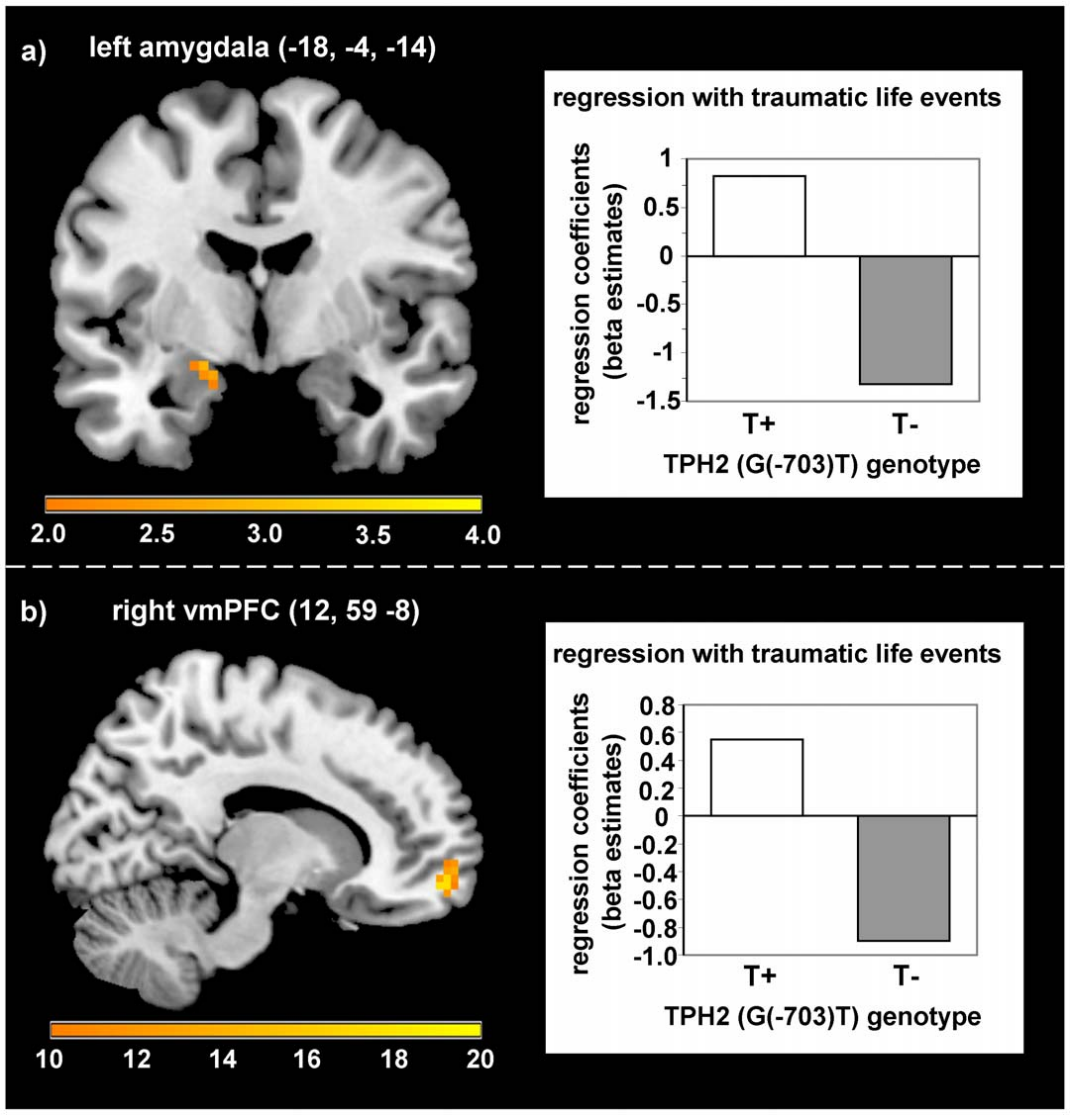
Modulation of neural activation by TPH2 (G(−703)T) polymorphism and traumatic life events. a) Stronger positive association of traumatic life events with left amygdala activation in T-allele carriers compared with non-carriers during late acquisition (*T* = 2.92, *p_fwe_* = .040). b) Stronger positive association of traumatic life events with right ventromedial prefrontal cortex activation in T-allele carriers compared with non-carriers during extinction (*F* = 17.59, *p_fwe_* = .016).

During late acquisition, a higher number of traumatic life events was positively correlated with differential responses (CS+ minus CS−) in the left amygdala (MNI: −18, −4, −14; *T*(66) = 2.92; *p_fwe_* = .040; Cohen’s *d* = .719) in T-allele carriers compared with non-carriers (see Figure 5a). No further interactions on (un)conditioned SCRs and neural responses emerged during early and late acquisition.

**Figure 6:**
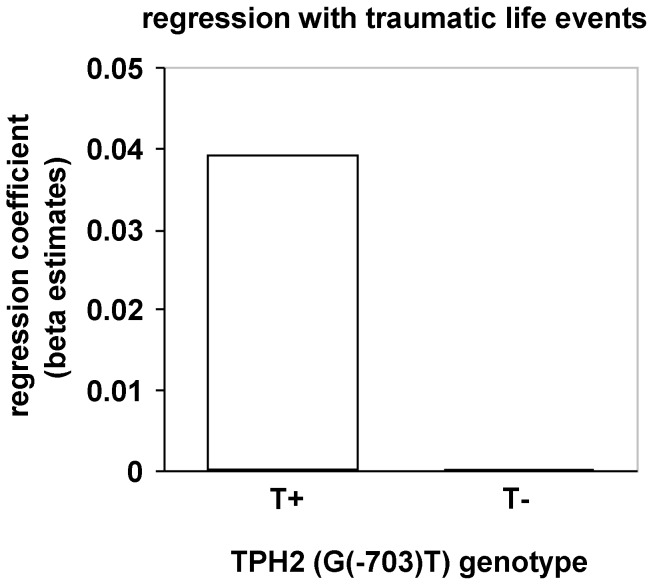
Modulation of conditioned skin conductance responses (SCRs) during extinction by TPH2 (G(−703)T) genotype and traumatic life events. Stronger positive association of conditioned SCRs during extinction in T-allele carriers compared with non-carriers.

During extinction learning an interaction with traumatic life events appeared in the right vmPFC (MNI: 12, 59, −8; *F*(1,66) = 17.59, *p_fwe_* = .016, Cohen’s *d* = 1.054), with a stronger positive association in T-allele-carriers compared with non-carriers (see Figure 5b). Furthermore, T-allele carriers with a higher number of traumatic events compared with non-carriers showed stronger first interval electrodermal responses during the extinction phase (*F*(1,62) = 5.90, *p* = .018; see Figure 6).

There appeared no further interactions with traumatic life events for (un)conditioned neural and skin conductance responses during all learning phases.

### Main Effect TPH2 (G(−703)T)

No main effect of TPH2 (G(−703)T) genotype emerged for conditioned and unconditioned neural and skin conductance responses.

## Discussion

The goal of the present study was to investigate the effects of serotonergic gene polymorphisms (5-HTTLPR and TPH2 (G(−703)T)) and their interaction with traumatic life events on neural and electrodermal correlates of fear acquisition and extinction in a large sample of healthy subjects.

Concerning the 5-HTTLPR, stronger insula activation was observed in S-allele carriers compared with non-carriers during late acquisition. Hyperactivation of this region is a typical finding during fear conditioning [5], [6] and processing of negative emotions in general [45], as well as during symptom provocation in anxiety disorders [6]. From a functional perspective, the insula seems to be especially important in perceiving and monitoring internal physical sensations [46] and in the anxious anticipation of aversive events [47], [48]. Altered autonomic functioning as recently reported for S-allele carriers compared with non-carriers (reduced vagal tone and a tendency for increased sympathetic activity; [19]) might be related to a stronger insula-mediated sensation of physiological arousal.

This enhanced conditioned responding in the insula is in line with previous findings showing increased conditioned insula activation [18] and increased conditioned startle responses [17] during fear conditioning in S-allele carriers compared with non-carriers. Regarding the finding that LL-homozygotes showed less intense conditioned responding in the insula compared with S-allele carriers one might speculate that this group shows reduced fear conditioning in this artificial experimental situation. However, that does not necessarily mean that LL-homozygotes exhibit impaired fear conditioning in relevant situations which have a stronger impact on their personal lives.

Importantly, in our study there were stronger unconditioned responses in S-allele carriers in the insula, too. One possible explanation might be a boosted acquisition in S-allele carriers due to stronger responding to the aversive UCS. This is in line with previous studies showing enhanced emotional processing in relevant brain regions in S-allele carriers compared with non-carriers [13], [14]. However, one previously published conditioning study did not find differences in neural UCS processing between S-allele carriers and non-carriers [18]. In this study aversive pictures instead of electrical stimulation were used as unconditioned stimuli, which might have contributed to differences in results compared with our study. The enhanced neural UCS processing in S-allele carriers in the insula was not paralleled by unconditioned skin conductance responses or intensities of electrical stimulation after calibration. This is in line with previous studies also showing no differences in unconditioned SCRs [17], [18] or intensity of electrical stimulation [17], [21] between genotype groups. A second possibility for stronger unconditioned responding in the insula in S-allele carriers in our study might be that enhanced activation during anticipation of the UCS lasts until the beginning of the electrical stimulation, and increases the unconditioned responding in this region. This might also contribute to the observed differences between studies and needs to be specifically tested in future studies clearly differentiating neural responses towards unconditioned stimuli from conditioned responses. Yet, the exact relation between these enhanced conditioned and unconditioned insula responses and its direction remains unclear. All in all, the enhanced unconditioned responding in the insula in this study is a critical point and makes it difficult to clearly interpret the increased conditioned responding in S-allele carriers in this brain structure.

In accordance with Lonsdorf and colleagues [17] and Klucken and colleagues [18], but in contrast to other studies [16], [19], we did not find an effect of 5-HTTLPR genotype on differential conditioned SCRs. This mismatch might be due to different methodological factors (e.g., sample selection, [16]; Pavlovian fear conditioning in our study vs. observational fear learning, [19]). However, we found enhanced differential SCRs (SIR) during late acquisition in S-allele carriers with a higher number of previously experienced traumatic life events compared with non-carriers, indicating that several risk factors (e.g., genetic and environmental) might be necessary to result in enhanced conditioned [peripheral] physiological responding. This is in line with a recent fear conditioning study showing enhanced conditioned responding in the insula and occipital cortex in SS-homozygotes with more stressful life events in the past [18]. However, interactions with environmental factors have been disregarded in other previous genetic fear conditioning studies, but might have contributed to the results unnoticed.

During extinction learning, an enhanced number of traumatic life events was associated with reduced responding in the left amygdala in carriers of the S-allele compared with non-carriers. This result corresponds with previous studies showing contrary effects of stressful life events in S-allele carriers vs. non-carriers on amygdala activation during the processing of e.g. negative [49] and neutral [13] facial stimuli. Rodent based models indicate an important role of the amygdala not only during fear acquisition but also during extinction learning [50]. Furthermore, there is evidence for distinct subpopulations of basolateral amygdala neurons differentially activated during fear vs. extinction learning [51]. Furthermore, amygdala activation is a common finding in human neuroimaging studies after the shift in CS-UCS contingency during extinction [8], [52], [53]. Additionally, higher amygdala baseline perfusion has been shown to be related to probably beneficial reduced dACC and enhanced vmPFC activation during extinction learning in human subjects [54]. The observed reduced amygdala activation in S-allele carriers with a higher number of TLEs might hence be interpreted as a dysfunctional neural responding during the formation of a new CS–no UCS association [50]. This could result in altered encoding and consolidation processes potentially leading to difficulties in retrieving the extinction memory at a later time point. This is in accordance with a previous study in patients with posttraumatic stress disorder (PTSD), showing stronger recurrence of symptoms 6 months after cognitive behavioural treatment in S-allele carriers compared with non-carriers [55], but no differences between genotype groups in the treatment response directly after therapy. This relapse might arise from difficulties retrieving the extinction memory in contrast to the original fear memory at this later point in time. The results of our study fit this clinical data very well and indicate that extinction mechanisms might further be influenced by the previous history of traumatic life events, potentially mediated by epigenetic mechanisms. However, other studies indicate that 5-HTTLPR genotype is not associated with treatment response in depressed patients [56] and patients with panic disorder [57]. One study even demonstrated enhanced treatment response in SS-homozygotic children with anxiety disorders compared with L-allele carriers at 6-month follow-up [58]. Taken together, these mixed results indicate the importance of future studies, in order to replicate these results and elucidate factors underlying the observed differences in modulation of treatment responses by 5-HTTLPR genotype.

Overall, our results regarding the serotonin transporter polymorphism suggest that carriers of the S-allele (with a higher number of previous traumatic life events) show indices of stronger fear acquisition or expression and an altered neural endophenotype during fear extinction, possibly leading to a higher risk to develop anxiety or mood disorders. However, as we investigated a sample of healthy young individuals the observed results might be affected by unknown resilience factors and thus prevents a clear interpretation of the data.

Concerning the TPH2 (G(−703)T) polymorphism, the results of our study show an association of a higher number of traumatic events with enhanced amygdala activation during late acquisition in T-allele carriers compared with non-carriers. This is in line with previous studies stressing the critical role of the amygdala in emotional processing of T-allele carriers [31], [32], fear acquisition in general [5], [6], and altered emotional processing in subjects with anxiety disorders [6]. However, this adverse or less beneficial effect on amygdala activation in T-allele carriers compared with non-carriers could only be observed, if they have previously been exposed to a higher number of traumatic life events, supporting the importance of considering gene-environment interactions in identifying neural endophenotypes.

During extinction learning we observed enhanced first interval SCRs in T-allele carriers with a higher number of traumatic life events compared with GG-allele carriers. This could be related to the observed stronger conditioned responses in the amygdala during acquisition and might indicate a dysfunctional prolonged conditioned fear expression during extinction. Correspondingly individuals with a higher number of traumatic life events in the T-group exhibited enhanced activation in the vmPFC compared with the GG-group. The vmPFC is a key structure involved in the acquisition and retrieval of extinction as indicated by findings in rat and human research, whereas a stronger recruitment of the vmPFC is associated with enhanced extinction memory (for an overview see [50]). Furthermore, subjects with posttraumatic stress disorder are characterized by a reduced activation of this region during recall of extinction [59]. Keeping in mind the stronger amygdala activation during late acquisition and enhanced SCRs during extinction, the enhanced vmPFC activity in T-allele carriers might be related to strengthened extinction learning due to a higher need to compensate stronger conditioned responding. It might be possible that these healthy participants are able to benefit from traumatic life events by learning and intensifying meaningful emotion regulation skills that might be advantageous to a certain extent in the future.

A central question is to what extent several genotypes interact in modulating the vulnerability for, or resilience against developing affective psychopathology. Accordingly, we examined the association of the combined genotypes with neural responses during fear conditioning. Although no differences were observed during the acquisition of emotional responses, extinction learning was accompanied by stronger dACC activation in subjects with a higher number of T+ and/or S+ genotypes. This result may indicate a prolonged fear expression, as the dACC has functionally and structurally been related to fear expression during classical conditioning [6], [60], [61], as well as to symptom provocation in several anxiety disorders [6]. The lack of increased dACC responses during acquisition seems inconsistent, but a possible explanation for this finding might be that a detrimental effect of the combined genotype on the dACC may consist in a prolonged fear expression despite extinction instead of enhanced acquisition or fear expression.

However, some limitations of the presented study need to be discussed: Despite having a relatively large sample size and a nearly equal distribution of participants over genotype groups (as compared with other studies in the field of genetic imaging), the sample size is still very small to reliably assess gene x gene interactions. Therefore the non-results of this interaction need to be interpreted with caution and should be further investigated in future studies with larger sample sizes.

In addition, it is unclear to which extent the results of our study can be translated to clinical populations and potentially help to better understand factors involved in the development and maintenance of mental disorders. As we studied healthy young individuals, unknown resilience factors might have contributed to the presented results and make it difficult to evaluate the clinical significance of our findings. Further (longitudinal) studies are needed to gain deeper insight into genetic and epigenetic mechanisms involved in the development of emotional disturbances and resilience.

Another major question left unanswered is, which biological mechanisms might underpin the observed group differences in fear learning and extinction. One might speculate that certain variants of the genetic determinants of serotonergic system activity might predispose a person to an increased reactivity to emotional stimuli. This might in orchestra with an exposition to an increased number of traumatic life events lead to modifications of the basal activity of the serotonergic system which might explain individual differences in emotional responsiveness. However, the underlying biological mechanisms need to be investigated in future studies. Thereby, studies trying to elucidate the potential epigenetic effects underlying the interaction of genetic variants and traumatic life events in detail might be especially fruitful. One possible strategy would be to analyze potential effects of traumatic life events on methylation patterns of the respective promoter regions of the *5-HTT* and *TPH2 genes* as well as a potential modulation of this association by 5-HTTLPR and TPH2 (G(−703)T) genotype.

In conclusion, we demonstrated a significant association between serotonergic gene polymorphisms and traumatic life events with neural responses during emotional learning in healthy subjects. These effects were apparent in the amygdala and insula during fear acquisition and in the amygdala, vmPFC and dACC during fear extinction. Altered activation in these structures during emotional learning might be a neural endophenotype, potentially translating genetic and adverse environmental factors into vulnerability for, or resilience against developing affective disorders.

## Supporting Information

Figure S1 Neural conditioned and unconditioned responses for the regions of interest in the whole group. All coordinates (x, y, z) are given in MNI space.
(DOC)
