# Supplementary material for: Functional Gene Polymorphisms in the Serotonin System and Traumatic Life Events Modulate the Neural Basis of Fear Acquisition and Extinction
Source: PLoS One. 2012 Sep 5;7(9):e44352. doi: 10.1371/journal.pone.0044352 (PMC3434167; doi:10.1371/journal.pone.0044352)
Supplement: Figure S1 — Neural conditioned and unconditioned responses for the regions of interest in the whole group. All coordinates (x, y, z) are given in MNI space. (DOC) [file pone.0044352.s001.doc]

S1

| Brain structure | **Side** | ***x*** | ***y*** | ***z*** | ***T _max_*** | ***p_FWE_*** |
| --- | --- | --- | --- | --- | --- | --- |
| **Early acquisition (CS+ minus CS-)** | | | | | | |
| insula | left | -36 | 14 | 4 | 8.69 | <.001 |
| insula | left | -36 | -19 | -2 | 4.00 | .010 |
| insula | right | 33 | 17 | 7 | 7.14 | <.001 |
| amygdala | left | -27 | -4 | -26 | 3.17 | .028 |
| amygdala | right | 24 | 2 | -23 | 5.13 | <.001 |
| dACC | - | 6 | 8 | 40 | 9.32 | <.001 |
| vmPFC | left | -15 | 14 | -14 | 5.69 | <.001 |
| vmPFC | right | 21 | 11 | -17 | 5.86 | <.001 |
| **Late acquisition (CS+ minus CS-)** | | | | | | |
| insula | left | -39 | 8 | 1 | 6.92 | <.001 |
| insula | right | 33 | 23 | 4 | 7.19 | <.001 |
| amygdala | left | -30 | -4 | -20 | 3.69 | .006 |
| amygdala | right | 30 | -1 | -26 | 5.62 | <.001 |
| dACC | - | 6 | 14 | 37 | 7.35 | <.001 |
| vmPFC | left | -18 | 11 | -14 | 4.99 | <.001 |
| vmPFC | right | 18 | 20 | -14 | 4.21 | .006 |
| **Extinction (CS+ minus CS-)** | | | | | | |
| insula | left | -33 | 20 | 4 | 3.57 | .033 |
| insula | right | 36 | 23 | -2 | 3.87 | .014 |
| dACC | - | -6 | 11 | 43 | 3.98 | .004 |
| **Unconditioned responses (UCS minus Non-UCS)** | | | | | | |
| insula | left | -39 | 5 | -2 | 20.77 | <.001 |
| insula | right | 39 | 5 | -2 | 23.08 | <.001 |
| amygdala | left | -24 | -1 | -14 | 11.30 | <.001 |
| amygdala | right | 27 | -1 | -14 | 10.94 | <.001 |
| dACC | - | 3 | 20 | 34 | 18.23 | <.001 |
| vmPFC | left | -18 | 11 | -14 | 4.43 | .003 |
| vmPFC | right | 21 | 11 | -14 | 7.51 | <.001 |
